# Elucidation of functional consequences of signalling pathway interactions

**DOI:** 10.1186/1471-2105-10-370

**Published:** 2009-11-06

**Authors:** Adaoha EC Ihekwaba, Phuong T Nguyen, Corrado Priami

**Affiliations:** 1 The Microsoft Research-University of Trento, Centre for Computational Systems Biology, Piazza Manci 17 Povo (Trento), Italy; 2 Dept. of Information Engineering and Computer Science, University of Trento, Trento, Italy

## Abstract

**Background:**

A great deal of data has accumulated on signalling pathways. These large datasets are thought to contain much implicit information on their molecular structure, interaction and activity information, which provides a picture of intricate molecular networks believed to underlie biological functions. While tremendous advances have been made in trying to understand these systems, how information is transmitted within them is still poorly understood. This ever growing amount of data demands we adopt powerful computational techniques that will play a pivotal role in the conversion of mined data to knowledge, and in elucidating the topological and functional properties of protein - protein interactions.

**Results:**

A computational framework is presented which allows for the description of embedded networks, and identification of common shared components thought to assist in the transmission of information within the systems studied. By employing the graph theories of network biology - such as degree distribution, clustering coefficient, vertex betweenness and shortest path measures - topological features of protein-protein interactions for published datasets of the p53, nuclear factor kappa B (NF-κB) and G1/S phase of the cell cycle systems were ascertained. Highly ranked nodes which in some cases were identified as connecting proteins most likely responsible for propagation of transduction signals across the networks were determined. The functional consequences of these nodes in the context of their network environment were also determined. These findings highlight the usefulness of the framework in identifying possible combination or links as targets for therapeutic responses; and put forward the idea of using retrieved knowledge on the shared components in constructing better organised and structured models of signalling networks.

**Conclusion:**

It is hoped that through the data mined reconstructed signal transduction networks, well developed models of the published data can be built which in the end would guide the prediction of new targets based on the pathway's environment for further analysis. Source code is available upon request.

## Background

"*Any classification in a division of objects into groups is based on a set of rules - it is neither true nor false (unlike, for example, a theory) and should be judged largely on the usefulness of the results*" [1].

For many years, model organisms have been studied extensively by scientists as they tried to better understand the functional implication of processes initiated during cellular signalling, and how organisms can use this to respond to perturbations outside of the cell [2]. With the advent of high throughput experimentation, the identification and characterization of molecular components involved in transduction events became possible in a systematic way. In addition to this, the discovered interactions between each of these components promoted the reconstruction of reactions leading to signaling pathways. Thus, elucidating the functional consequences of these interactions will be crucial in understanding the ways in which cells respond to extra cellular cues and how they communicate with one another.

Activities of biological cells are regulated by proteins carrying signals that modify the expression of different genes at any given time, and these extra-cellular signals drive cell proliferation and programmed cell death via complex signal transduction circuits comprising of receptors, kinases, phosphatases, transcription factors and many others. It is unsurprising that many components of these signal transduction circuits are oncogenes or tumour suppressors, emphasizing the importance of understanding signalling in normal tissues and targeting aberrant signalling in diseases [3]. Signalling networks which are chiefly based on interactions between proteins are the means by which a cell converts an external signal (*e.g. stimulus*) into an appropriate cellular response (*e.g*. cellular rhythms - periodic biological process observed in cell cycles or day-night cycles (circadian rhythms) of animals and plants) [4-6]. It is from the resulting basic cellular responses that complex behaviour in multi-cellular organisms emerges.

Signal transduction pathways have typically been drawn as separate linear entities, however it has become increasingly clear that signalling pathways are extensively interconnected and are embedded in networks with common protein components and cross talk with other networks [7-11]. In addition to this, signal transduction networks do not depend merely on the shifting of relevant protein concentrations from one steady state level to another, rather, the signals often have a significant temporal variation that carries much more information that is propagated in a complex manner through the networks [12-15].

Traditionally, study of the complex behaviour of networks require dynamic models that contain both the biochemical reactions as well as their rate constant counterparts [16-19]. This information is usually not accessible directly through experiments for systems less well studied. Fortunately for many biological systems partial prior knowledge about the connectivity patterns of the networks is becoming available and readily stored in databases [20-23], even though the detailed mechanisms still remain undiscovered. An important goal of this research therefore is to attain a reconstruction of the network of interactions that gives rise to signalling pathways in a biologically meaningful way, which in turn allows the mathematical analysis of the emerging properties of the network [24,25].

So far, a great deal of data has accumulated on signalling systems and these large datasets are thought to contain much information on the structure of their underlying networks. However, this information is hidden and requires advanced algorithms and methods, such as data mining and graph theories of network biology to make sense of it all [26-28]. Data mining deals with the discovery of hidden knowledge, unexpected patterns and new rules [29]; nevertheless, there are some limitations with this technique. A fundamental issue is that biological data repositories are normally presented in heterogeneous and unstructured forms [30-33]. Therefore, there is a great need to develop effective data mining methodologies to extract, process, integrate and discover useful knowledge from multiple data sources [34]. The retrieved knowledge can then be better organized and structured to develop models, which in the end, would guide the prediction of new targets based on the pathway's environment [24,26-28,35,36].

In this report, we present a systems analysis framework to examine how protein-protein interactions within these systems relate to multi-cellular functions, and how high throughput technologies allow the study of the different aspects of signalling networks for modelling. We assume that since mammalian cells are constantly remodelling their transcriptional activity profiles in response to a *combination *of inputs, the understanding of their coordinated responses have been lacking, and in essence requires a framework which examines the system or systems by extracting information on their topological and functional properties. An example of a system activated in response to a variety of signals is the NF-κB pathway [19,37-40] (a family of proteins which functions as DNA-binding proteins and transcription factors); the disruption of which in recent years have been shown to contribute towards the many human diseases presently known. We also know from literature [41-43] that the NF-κB network does not exist in isolation, since many of its mechanisms have been shown to integrate their activity with other cell signalling networks. Such as the p53 system [17,44-49] (another transcriptional activator that plays an important role in the regulation of apoptosis) and the E2F-1 [50-53] - a cell cycle transcriptional target that controls the expression of a number of genes needed for DNA synthesis and progression into S phase [46,49,54-59]. It is thought that the cooperation between p53, NF-κB and E2F-1 is most likely to reflect on their ability to function together to induce expression of target genes regulated by promoters containing p53, NF-κB and E2F-1 binding sites [53,60,61], since target genes translated to proteins in one way or another affect the individual system in a positive or negative way.

To capture the possible events involved in the pathways, *only proteins involved in the oscillatory feedback loops of the systems were considered *- which are ubiquitous feature of the biological examples given which can be adapted to yield distinct system level properties [16,17,40,62]. To generate the networks, the molecular components and their interactions were extracted from publicly available datasets [20-23]. In addition, associations of these networks with some cell cycle proteins, in particular, the G1/S phase cell cycle proteins [63,64] were also examined. Cell cycle proteins were considered since previously published literature showed some of its proteins to be activated by one pathway and to be relevant for the regulation of another [44,65-70]; and thus may be useful in showing a level of complexity not visible by looking at the NF-κB and p53 systems alone. We next identified key nodes of significant influence in the isolated systems investigated using some graph theories of network biology, namely, degree, vertex betweenness, and clustering coefficient measures. We used shortest paths calculation to find connecting nodes, most likely responsible for the propagation of transduction signals across the networks. And cross referencing them with reference databases, the interpretation of the functional properties of these key nodes, as well as, the highly ranked connecting nodes within the systems were realised. The idea is that through the data mined reconstructed signal transduction pathways which are comparable to the previously modelled networks of the real system, a phenomenological model of all the published data can be derived from which the key components of the system can be highlighted for further analysis. In fact, as we will show in this report, it is possible to reconstruct signalling networks in this way without additional constraint.

## Methods

**Figure 1:**
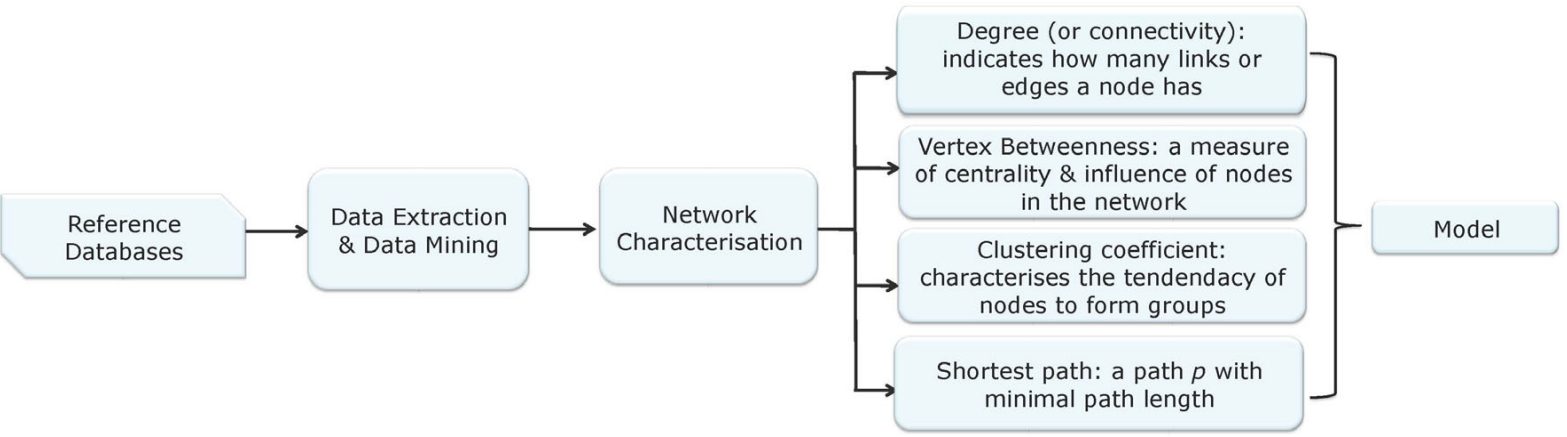
**A Schematic representation of the modelling framework introduced**.

The development of high-throughput molecular assay technologies, as well as breakthroughs in information processing and storage technologies provide integrated views of biological and medical information. Databases enabling systematic data mining on bio-molecular interactions, pathways and molecular disease associations are becoming increasingly available, which it is hoped will facilitate the understanding of the dynamics of biological function in complex diseases. Summarised below are descriptions of the analytical methods used in this study - see Figure 1 for a schematic representation of the framework.

### Definition of Reference Databases

Over the last few years many of the experimental data from gene expression studies have been made freely available for academic research in the form of reference databases [20-23] of which several exist. These different databases have their strengths and weaknesses and there is no universal method best for storing these data sets. A number of different approaches have been used to extract signalling data and integrate them for biologically valid conclusions to be drawn from the vast and comprehensive data sets available [71,72]. Table 1 lists a description of the individual databases used in this study, each of which was used to retrieve information related to the proteins considered. These databases contain information on proteins, protein interactions and biological processes.

**Table 1:** Reference databases used for data retrieval during the investigation

*Database*	*Description*	*URL*	*Statistics*	*Data extracted*
Uniprot [20]	comprehensive, high-quality and freely accessible resource of protein sequence and functional information.	http://www.uniprot.org	220,325 entries	function,, post-translation modification, location, developmental stage, etc.
I2d [21]	on-line database of known and predicted mammalian and eukaryotic protein-protein interactions	http://ophid.utoronto.ca/	424,066 entries (92,561 for human)	protein interaction
Reactome [22]	curated resource of core pathways and reactions in human biology.	http://www.reactome.org	928 pathways for human	Pathway
PID [23]	curated pathway and interactions	http://pid.nci.nih.gov/	133 pathways	Pathway

### Data extraction and data-mining

The concerted efforts of genetics, molecular biology, biochemistry and physiology have led to the accumulation of an enormous amount of data on molecular components of signalling networks reported in the literature or stored in databases [73]. The availability of these vast amounts of data provides an opportunity for investigating further the design principles underlying structure and dynamics of signalling networks [71,72,74]. However, these data are diverse and dispersed in different databases. For this reason, data mining is employed and takes the responsibility of mining this amount of data in the hope that it will return useful hypotheses supporting life sciences. Due to its capability of processing different kinds of data, data mining has the ability to integrate these spread-out data in a unified framework thus solving more efficiently the problems that may arise due to their differences [29,30,32].

We started by looking into four databases: Universal Protein Resource (*Uniprot*), Interologous Interaction Database *(i2d)*, *Reactome *and Pathway Interaction Database (*PID*), which we have listed in Table 1. Since different databases have different names for each entry, the *Uniprot *name for identifying proteins was used as the standard and thus all protein names were converted accordingly to their *Uniprot *counterparts. In addition, in the *Uniprot *database, protein information is published for a wide-range of organisms and curated from different sources. A search for p53 in *Uniprot *returns 1,624 results, such as [Uniprot:P04637] (P53_HUMAN) for human, [Uniprot:P02340] (P53_MOUSE) for mouse, [Uniprot:P13481] (P53_CERAE) for green monkey. To assure the proteins extracted from *Uniprot *are the exact proteins from the organism of interest, a form of verification was implemented, where the identity of the mined data is confirmed through a form of literature search. This step avoids the confusion and ambiguity that often occurs when mining and integrating multiple data. Table 2 lists the search proteins considered in the study (highlighted proteins are proteins reported to be activated in one system and involved in the regulation of another).

**Table 2:** Proteins and pathways considered in the study

Network	Uniprot accession	Uniprot entry name	Alternative name
p53 pathway	P04637	P53_HUMAN	p53
	Q00987	MDM2_HUMAN	mdm2
	P38936	CDN1A_HUMAN	**p21**
	Q8N726	CD2A2_HUMAN	**p14ARF**
NF-κB pathway	O00221	IKBE_HUMAN	NF-κB inhibitor epsilon
	O14920	IKKB_HUMAN	IKK2
	O15111	IKKA_HUMAN	IKK1
	P19838	NFKB1_HUMAN	Nuclear factor NF-κB p105 subunit
	P25963	IKBA_HUMAN	IκB-alpha
	Q00653	NFKB2_HUMAN	Nuclear factor NF-κB p100 subunit
	Q01201	RELB_HUMAN	Transcription factor RelB
	Q04206	TF65_HUMAN	Transcription factor p65 (RelA)
	Q04864	REL_HUMAN	C-Rel protein
	Q14164	IKKE_HUMAN	Inhibitor of nuclear factor κB kinase subunit epsilon
	Q15653	IKBB_HUMAN	NF-kappa-B inhibitor beta
	Q96HD1	CREL1_HUMAN	Crel1
	Q6UXH1	CREL2_HUMAN	Crel2
	Q9Y6K9	NEMO_HUMAN	IKKγ
G1/S phase cell	P24385	CCND1_HUMAN	Cyclin D1
cycle proteins	Q01094	E2F1_HUMAN	E2F-1
	P06400	RB_HUMAN	Rb
	P46527	CDN1B_HUMAN	P27

The proteins have been listed according to their *Uniprot *accession names.

Using the *i2d *database, information on protein-protein interactions was extracted. Such information is potentially useful in identifying proteins and their families, the interplay with their interacting partners, the influence of certain proteins in a network and key regulatory relationships which are most influenced by extracellular signals. More comprehensive knowledge concerning the proteins of interest and their connector proteins, for example, biological process, cellular component, coding sequence diversity, developmental stage, disease, domain, ligand, molecular function and post-translation modification were also extracted. For elucidating the functional consequences of the interactions, the *Reactome *database - which gives pathway information by combining with graph information of the *PID *database - was the database of choice. Table 3 presents a list of pathways and/or processes the explored proteins were revealed to be involved in. The data mining implementation was done in Perl programming language http://www.perl.org/ and derived from BioPython library http://biopython.org/wiki/Main_Page.

**Table 3:** Pathways and biological processes information retrieved from *Reactome *database

Uniprot accession	Uniprot entry name	Pathway
P25963	IKBB_HUMAN	[2 processes]: Signalling in Immune system; Signalling by NGF
Q15653	REL_HUMAN	Signalling in Immune system
O15111	IKKB_HUMAN	[2 processes]: Signalling in Immune system; Signalling by NGF
O14920	IKKE_HUMAN	[2 processes]: Signalling in Immune system; Signalling by NGF
P19838	IKBA_HUMAN	[2 processes]: Signalling in Immune system; Signalling by NGF
Q00653	IKBE_HUMAN	Signalling in Immune system
Q04206	IKBZ_HUMAN	[2 processes]: Signalling in Immune system; Signalling by NGF
P04637	P53_HUMAN	Cell Cycle Checkpoints
P38936	CDN1A_HUMAN	[3 processes]: Cell Cycle Checkpoints; Cell Cycle, Mitotic; DNA Replication
Q00987	MDM2_HUMAN	[2 processes]: Cell Cycle Checkpoints; Signalling by NGF
P46527	CDN1B_HUMAN	Signalling by NGF

*Reactome *can either be directly browsed or queried by text search using, for instance using *UniProt *accession numbers, to identify events or pathways considered search proteins are involved in.

### Network Biology

The actions of specific proteins in a network have been investigated in this report. A network can be described as a series of nodes/vertices that are connected to each other by links. Formally it was referred to as a graph and the links as edges [26,75-77]. The nodes in biological networks are the gene products/proteins and the links the interactions between two components [13,78]. A number of metrics have been used to characterise the networks of the systems studied:

• The first, the degree (or connectivity) of a node/vertex *k*, indicates how many links/edges the node has to the other nodes. Of particular importance is the degree distribution *P(k)*, which measures the probability that a selected node has exactly *k *links. The degree distribution is used to distinguish between the different classes of network (which has not been reported in this account).

• The second, vertex betweenness (*B*_*i*_) is a measure of the centrality and influence of nodes in the networks [79-82].

• The third, average clustering coefficient *C(k)*, characterises the overall tendency of nodes to form clusters or groups; and *C(k) *the average clustering coefficient of all nodes with *k *links is an important measure of the network structure [15].

• And finally, the shortest path, which is found between two vertices (or nodes) such that the sum of the weights of its constituent edges is minimized [82,83].

A graph *G(E, V) *consists of a set of vertices *(V) *and a set of edges *(E) *between them. An edge *e*_*ij *_connects vertex *v*_*i *_with vertex v_j_. Here, undirected graph is investigated since our studied protein interaction networks are undirected. An undirected graph has the property that *e*_*ij *_and *e*_*ji *_are considered identical. Therefore, the neighbourhood N for a vertex *v*_*i *_is defined as it's immediately connected neighbours in Eq. (1):(1)

where the degree *k*_*i *_of a vertex is defined as the number of vertices |*N*_*i*_|, in its neighbourhood *N*_*i*_.

The betweenness centrality of a vertex *v*_*i *_is defined as the number of shortest paths between pairs of other vertices that run through *v*_*i *_as Eq. (2):(2)

where *i *≠ *j *≠ *k*, *g*_*jk *_is the number of equally shortest paths between nodes *v*_*j *_and *v*_*k*_, and *g*_*jk*_*(i) *the number of the shortest paths where node *v*_*i *_is located [84].

The clustering coefficient *C*_*i *_for a vertex *v*_*i *_is given by the proportion of links between the vertices within its neighbourhood divided by the number of links that could possibly exist between them [15]. Therefore, if a vertex *v*_*i *_has *k*_*i *_neighbours, *k*_*i*_*(k*_*i*_*-1)/2 *edges could exist among the vertices within the neighbourhood where the clustering coefficient for undirected graphs can be defined as Eq.(3):(3)

For the shortest path, given a real-value weight function *f*: *E *→ **R**, and a start node *v*_*i *_of *V*, we find a path *p *of *P *(the set of paths) from *v*_*i *_to each *v*_*j *_of *V *if present (Eq. (4), so that(4)

**Figure 2:**
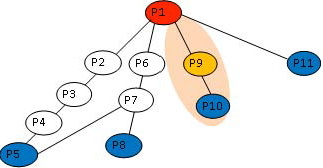
**Diagram of the shortest path calculation**. An Illustration showing how the shortest path discussed in the report is calculated. It is assumed that; from P1 to P5: *p*_1 _= (P1-P6-P7-P5) and *l*_1 _= 3. From P1 to P8: *p*_2 _= (P1-P6-P7-P8) and *l*_2 _= 3. From P1 to P10: *p*_3 _= (P1-P9- P10) and *l*_3 _= 2. From P1 to P11: *p*_4 _= (P1-P11) and *l*_4 _= 1.

If the protein-protein interaction networks here constitute an unweighted graph, the weight function *f *can be considered as a path length *l *(the number of edges in path *p*). In this case, the shortest path problem is to find a path *p *having the minimal path length. A Breadth-First Search algorithm [82,83] has been employed to find the shortest paths between two nodes (the starting node *v*_*i *_and destination node *v*_*j*_) (see Figure 2). The shortest paths may have different path lengths (*l *= 1, *l *= 2, *l *= 3, *l *= 4, etc.). In the example shown in Figure 2, there are different shortest paths from start node *P1 *to destination nodes (*P5, P8, P10, P11*) *via *different connector nodes (*P6, P7, P9*). If the path length is 1, this signifies a direct connection, where two nodes are directly connected (*e.g*., *P1 and P11*). For the shortest paths with *l *= 2, there are three nodes: a start node (*P1*), a connector node (*P9*), and a destination node (*P10*). Using this form of analysis the path lengths were used to obtain knowledge on the functional interactions between the proteins. For the purpose of this report we will only discuss findings for the shortest paths between two nodes of interest with path length *l *= 1 or *l *= 2; their connector nodes and their frequency ranking (*f*_*i*_) [see Additional file 1: Suppl. 1-5 for the full list of shortest paths with other path lengths]. A node is said to have a high frequency if there is an increase in the number of paths passing through it; thus a high frequency node may be the centre of the networks' cross talk. For a given set of 2-length shortest paths [26,85] {*p*_1_, *p*_2_, *p*_3_, ... *p*_*n*_} between two sets of nodes *V*_*i *_and *V*_*j*_, with *v*_1_, *v*_2_, *v*_3 _as connectors of those paths, if frequency *f*_1 _of *v*_1 _= 2 (if *v*_1 _is the connectors of two paths); *f*_2 _= 10 (if *v*_2 _is the connector of ten paths); *f*_3 _= 5 (if *v*_3 _is the connector of five paths); then the results of the ranking is *v*_2 _- *v*_3 _- *v*_1_. The highly ranked nodes obtained, is then suggested to be the most important nodes within the network.

Network biology computation was implemented in CoSBiLab-Graph http://www.cosbi.eu/index.php/research/prototypes/overview. CoSBiLab-Graph is a tool suitable for a variety of tasks on graphs like construction, visualisation, and modification. CoSBiLab-Graph can be used to calculate measures, run algorithms and layout graphs. The network visualisation is performed by the software NAViGaTOR ((Network Analysis, Visualization, & Graphing TORonto)) http://ophid.utoronto.ca/navigator/. NAViGaTOR is the add-in software package of *i2d *database, and thus supports the simulation of the protein interaction networks extracted from *i2d *in this report. Other network analysis tools used are Social Network Analysis Software http://www.analytictech.com/, and Centralities in Biological Networks http://centibin.ipk-gatersleben.de/.

## Results and Discussion

Recognising that individual signalling pathways do not act in isolation, an integrated approach to investigate the dynamic relationships between components, their organisation and regulation in signalling systems was undertaken. We started by searching the *i2d *database (containing *92,561 *human protein interactions) for the proteins of interest. This search retrieved a total of 1,881 protein-protein interactions for components of p53 and NF-κB networks (see Table 2). To increase the confidence in the extracted interactions information, we excluded 47 interactions shown to have been derived from other organisms (other than human) by homologous methods, so that the number of protein interactions obtained involving both the NF-κB and p53 networks consists of 1,834 interactions. Information on protein-protein interactions within the NF-κB and p53 pathways were also retrieved and analysed. Finally, the interlinking connections between the NF-κB and p53, and proteins involved in the G1/S phase of the cell cycle (in particular, RB_HUMAN, CCND1_HUMAN, CDN1B_HUMAN, CD2A2_HUMAN, E2F1_HUMAN and CDN1A_HUMAN) were also investigated (see Table 4 for statistical information retrieved for the networks).

**Table 4:** Statistical information on the nodes and interactions retrieved for the networks

*Network*	*Number of nodes*	*Number of interactions*	*Number of articulation points*
p53	436	506	7
NF-κB	788	1352	15
Cell cycle - Cyclin D1, Rb, E2F-1, p27	527	299	4
NF-κB and p53	1105	1834	18
NF-κB, p53 and Rb, E2F-1	1208	2032	20
NF-κB, p53 and Cyclin D1, Rb, E2F-1, p27	1239	2127	22

### Network of Interactions

**Figure 3:**
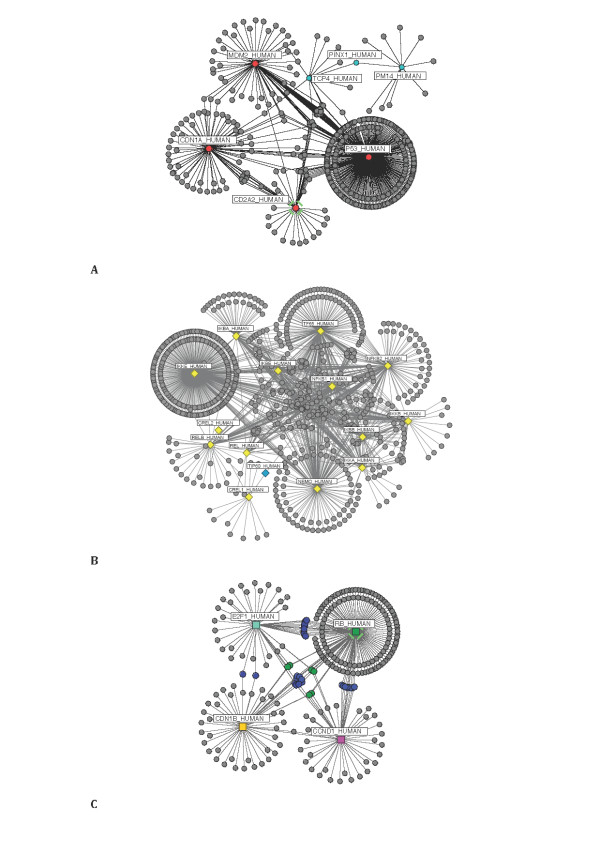
**Network representation of isolated p53, NF-κB and cell cycle systems**. A graphical representation of the (**A**) p53, (**B**) NF-κB, and (**C**) the G1/S transition phase of the cell cycle {RB_HUMAN, E2F1_HUMAN, CDN1B_HUMAN and CCND1_HUMAN} networks. The proteins are represented in the form of nodes, and their interactions in the form of edges. For the cell cycle network (**C**), the *shared components *linking RB_HUMAN, E2F1_HUMAN, CDN1B_HUMAN and CCND1_HUMAN to one another are highlighted (in green), and are six in number (*i.e*. three pairs). RB_HUMAN, CCND1_HUMAN and CDN1B_HUMAN connect with each other by CDK4_HUMAN and CDK2_HUMAN. RB_HUMAN, E2F1_HUMAN and CDN1B_HUMAN are linked together by CCNA1_HUMAN and SKP2_HUMAN. And finally RB_HUMAN, CDN1B_HUMAN, E2F1_HUMAN and CCND1_HUMAN link up with BRCA1_HUMAN and SP1_HUMAN as their connecting components.

Following data extraction, descriptive analysis of the data was performed. The degree, betweenness and cluster coefficient values for the network's components were calculated in order to ascertain the level of connectivity of the three systems. Figure 3 illustrates the molecular interactions obtained for the NF-κB, p53 and the G1/S phase cell cycle, respectively. Figure 3A and Table 4 show for the proteins in the p53 network, 506 interactions and 436 nodes. Seven of which are articulation points (four original search nodes (in red) and three other associated nodes obtained from the extraction process (in cyan)). Articulation nodes (or cut vertex) [86,87] are nodes that play an important role in a network, where the removal of the node may drastically alter the network topology leading to it's fragmentation. Conversely, for the NF-κB network (see Table 4 & Figure 3B) 788 nodes and 1,352 interactions were observed. The articulation points were fifteen in number, fourteen of which were the search proteins considered (in yellow) and an associated TIP60_HUMAN (in cyan) obtained during the extraction process. A subset of the highest connectivity or degree values are shown in Table 5 and 6 [see Additional file 1: Suppl. 6-10 for connectivity values obtained for nodes not included in the Tables]. We found that for the three networks examined, the calculated degree for the initial list of proteins, with the exception of the CREL2 protein in the NF-κB network (Table 2), were discovered to be much higher than the associated proteins found during the mining process; and therefore underscored the central role of the initial list within their individual networks (search proteins highlighted on Table 5 and 6; please note other nodes - TIP60_HUMAN in the NF-κB network (Figure 3B), and TCP4_HUMAN, PINX1_HUMAN and PM14_HUMAN in the p53 network (Figure 3A) - are associated articulation points).

**Table 5:** Degree and clustering coefficient values calculated for the p53 and NF-κB networks

*Network p53*				*Network NF-κB*			
**Uniprot accession**	**Uniprot entry name**	**Degree**	**Clustering coefficient**	**Uniprot accession**	**Uniprot entry name**	**Degree**	**Clustering coefficient**

**P04637**	**P53_HUMAN**	**300**	**9.10E-04**	**Q14164**	**IKKE_HUMAN**	**324**	**1.90E-04**
**Q00987**	**MDM2_HUMAN**	**72**	**0.0133**	**Q04206**	**TF65_HUMAN**	**186**	**0.01796**
**P38936**	**CDN1A_HUMAN**	**72**	**0.00509**	**Q9Y6K9**	**NEMO_HUMAN**	**157**	**0.01764**
**Q8N726**	**CD2A2_HUMAN**	**43**	**0.00664**	**Q00653**	**NFKB2_HUMAN**	**145**	**0.03218**
P53999	TCP4_HUMAN	12	0	**P19838**	**NFKB1_HUMAN**	**118**	**0.04578**
Q9Y3B4	PM14_HUMAN	10	0	**P25963**	**IKBA_HUMAN**	**85**	**0.05546**
P49459	UBE2A_HUMAN	3	0.66667	**O14920**	**IKKB_HUMAN**	**75**	**0.07279**
Q16665	HIF1A_HUMAN	3	0.66667	**Q15653**	**IKBB_HUMAN**	**73**	**0.06963**
P06748	NPM_HUMAN	3	0.66667	**O15111**	**IKKA_HUMAN**	**71**	**0.08089**
P25490	TYY1_HUMAN	3	0.66667	**Q01201**	**RELB_HUMAN**	**64**	**0.06399**
P62988	UBIQ_HUMAN	3	0.66667	**O00221**	**IKBE_HUMAN**	**48**	**0.11702**
P51959	CCNG1_HUMAN	3	0.66667	**Q04864**	**REL_HUMAN**	**38**	**0.14794**
Q92793	CBP_HUMAN	3	0.66667	**Q96HD1**	**CREL1_HUMAN**	**12**	**0.68182**
P62081	RS7_HUMAN	3	0.66667	P07437	TBB5_HUMAN	12	0.68182
Q99816	TS101_HUMAN	3	0.66667	P62158	CALM_HUMAN	12	0.68182

The table is arranged in descending order. Only the first fifteen proteins within the network with high degree values are listed.

**Table 6:** Degree and clustering coefficient values calculated for the cell cycle network

Uniprot accession	Uniprot entry name	*Degree*	*Clustering coefficient*
**P06400**	**RB_HUMAN**	**156**	**0.00232**
**P46527**	**CDN1B_HUMAN**	**50**	**0.00245**
**P24385**	**CCND1_HUMAN**	**48**	**0.01330**
**Q01094**	**E2F1_HUMAN**	**48**	**0.01418**
Q13309	SKP2_HUMAN	3	0.33333
P78396	CCNA1_HUMAN	3	0.33333
P08047	SP1_HUMAN	3	0.66667
P24941	CDK2_HUMAN	3	0.66667
P38398	BRCA1_HUMAN	3	0.66667
P11802	CDK4_HUMAN	3	0.66667
P20248	CCNA2_HUMAN	2	0.00000
Q9NQX5	NPDC1_HUMAN	2	0.00000
P00519	ABL1_HUMAN	2	0.00000
P33993	MCM7_HUMAN	2	0.00000
P30281	CCND3_HUMAN	2	0.00000

The highest-degree node (or connectivity) uncovered for the NF-κB network was IKKE_HUMAN, a protein responsible for inhibiting the NF-κB inhibitory subunits with 324 interactions (see Table 5) [88]. A discovery that suggests IKKE_HUMAN to be the most studied protein of the NF-κB system; and maybe a possible molecular target for therapy in the NF-κB system. In addition to this, four other proteins were found to have interacting proteins numbering over 100. These were: TF65_HUMAN (RelA), NEMO_HUMAN (IKKγ), NFKB2_HUMAN (p52), and NFKB1_HUMAN (p50) [Note - this finding could also be a reflection of the fact that these proteins may be the most studied members of the NF-κB network]. For the cell cycle network, the highly connected nodes were four in number (see Figure 3C and Table 4). Compared to the NF-κB network (Figure 3B), the p53 (Figure 3A) and the cell cycle (Figure 3C) networks appeared to be sparse, with each node connected to a relatively small number of edges within the network, many of whom "know" each other. The sparse nature could be explained by the fact that only proteins involved in the oscillatory feedback loops of the systems of interest, and not the entire published members were considered in this study. The highest-degree node for the p53 network was the P53_HUMAN protein, and RB_HUMAN for the selected cell cycle proteins (both with degree connectivity value's, 300 and 156 respectively - Table 5 and 6); a result signifying their importance in their various networks. Similarly, the vertex betweenness measure [80] also confirms IKKE_HUMAN, P53_HUMAN and RB_HUMAN as prominent nodes in their networks (Table 7) [see Additional file 1: Suppl. 11-13 for results obtained from other centrality measures]. In addition, various highly interconnected subgroups were also uncovered, namely: P53_HUMAN with MDM2_HUMAN; RELB_HUMAN with NFKB2_HUMAN; and E2F1_HUMAN with RB_HUMAN [see Additional file 1: Suppl. 1-3]. These subgroups could also be described as network *motifs *[89-91], frequently recurring groups of interactions, usually highly conserved, which are thought to perform specific information processing roles in the networks; in some cases supporting their roles as oscillators [5,18,63,92].

**Table 7:** Vertex betweenness values calculated for p53, NF-κB and cell cycle networks

*Network p53*			*Network NF-κB*			Cell Cycle		
**Uniprot accession**	**Uniprot entry name**	***B*_*i*_**	**Uniprot accession**	**Uniprot entry name**	***B*_*i*_**	**Uniprot accession**	**Uniprot entry name**	***B*_*i*_**

**P04637**	**P53_HUMAN**	**81612.87**	**Q14164**	**IKKE_HUMAN**	**166771.25**	**P06400**	**RB_HUMAN**	**26218.55**
**P38936**	**CDN1A_HUMAN**	**22279.58**	**Q04206**	**TF65_HUMAN**	**70319.46**	**P24385**	**CCND1_HUMAN**	**9196.14**
**Q00987**	**MDM2_HUMAN**	**18352.39**	**Q9Y6K9**	**NEMO_HUMAN**	**60543.29**	**Q01094**	**E2F1_HUMAN**	**8081.61**
**Q8N726**	**CD2A2_HUMAN**	**9223**	**Q00653**	**NFKB2_HUMAN**	**48010.40**	**P46527**	**CDN1B_HUMAN**	**7318.69**
P53999	TCP4_HUMAN	6801.47	**P19838**	**NFKB1_HUMAN**	**40763.53**	P24941	CDK2_HUMAN	1481.39
Q96BK5	PINX1_HUMAN	4250	**P25963**	**IKBA_HUMAN**	**40680.12**	P11802	CDK4_HUMAN	1481.39
Q9Y3B4	PM14_HUMAN	3870	**Q15653**	**IKBB_HUMAN**	**21318.82**	Q13309	SKP2_HUMAN	1195.79
P68400	CSK21_HUMAN	1706.11	**Q01201**	**RELB_HUMAN**	**17557.99**	P78396	CCNA1_HUMAN	1195.79
P20226	TBP_HUMAN	1607.64	**O14920**	**IKKB_HUMAN**	**15356.68**	P38398	BRCA1_HUMAN	872.01
Q09472	EP300_HUMAN	1607.64	**O15111**	**IKKA_HUMAN**	**14387.00**	P08047	SP1_HUMAN	872.01
P41235	HNF4A_HUMAN	1192.02	**O00221**	**IKBE_HUMAN**	**12109.01**	Q9Y3I1	FBX7_HUMAN	614.12
P12004	PCNA_HUMAN	514.08	Q92993	KAT5_HUMAN	8503	Q00526	CDK3_HUMAN	614.12
P21675	TAF1_HUMAN	415.62	**Q96HD1**	**CREL1_HUMAN**	**7785**	P20248	CCNA2_HUMAN	614.12
P06748	NPM_HUMAN	363.33	**Q04864**	**REL_HUMAN**	**6563.01**	P30281	CCND3_HUMAN	614.12
P08238	HS90B_HUMAN	363.33	P07437	TBB5_HUMAN	3982.81	P30279	CCND2_HUMAN	614.12

Results obtained by vertex betweenness produced similar results to the degree of connectivity index reported in Tables 5 and 6.

Following the characterisation of the three networks with respect to their degree of connectivity, further calculations were made on their clustering coefficients. It was discovered that MDM2_HUMAN (mdm2) in the p53 network, REL_HUMAN (C-Rel) in the NF-κB network and E2F1_HUMAN (E2F-1) of the cell cycle were proteins found to have the highest clustering coefficient values; a finding reflecting on the nodes connectivity within their neighbourhood. That is to say, even though P53_HUMAN, RB_HUMAN and IKKE_HUMAN were found to be proteins with the most interaction within their individual networks; MDM2_HUMAN, REL_HUMAN (C-Rel) and E2F1_HUMAN were revealed to be proteins best at forming cliques in their networks.

Having discovered for each system, the highly connected nodes, as well as the nodes with the most number of neighbours, it was of interest to study how all the individual system studied relates to each other. In order to do this, we set out to calculate the shortest paths and the frequency of proteins linking the systems to one another; thereby identifying key connector proteins thought to assist in the transmission of information (or cross talk) across the three networks. It was hoped that through this form of analysis, characteristics of the connector proteins linking the systems will be uncovered.

### Network of interactions between p53 and NF-κB pathways

**Figure 4:**
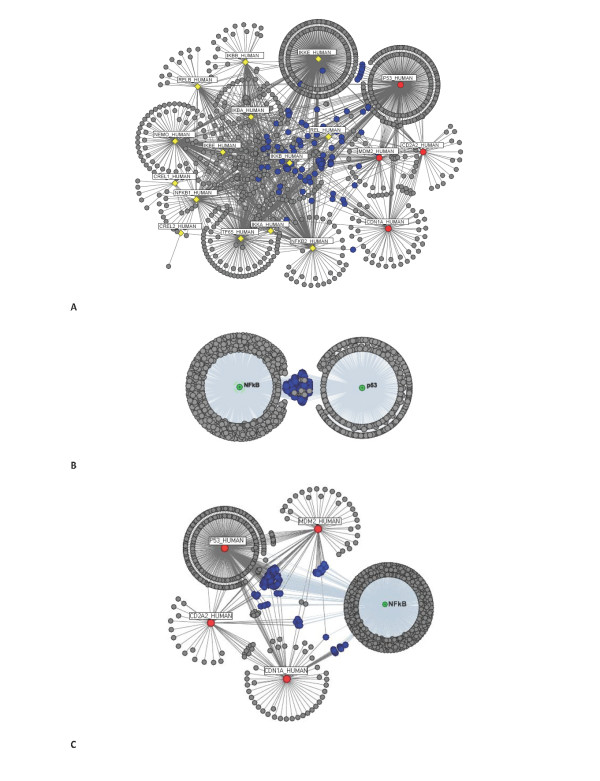
**A system of p53 and NF-κB**. A unified network of the (**A**) p53 (red circles) and NF-κB (yellow diamonds) networks, with their shared components clearly defined (in blue). (**B**) Condensed view of the two networks; and in (**C**) only the NF-κB network, which allows for a better visualisation of the connections.

Since it has been suggested that the topology of a network affects the spread of information carried by a signal and thus diseases [34], the network of interactions between the p53 and NF-κB systems were investigated. Figure 4 illustrates the complex network formed between the p53 and the NF-κB systems, and the connector proteins linking them (proteins in the p53 network are denoted in red, and those of the NF-κB are in yellow - Figure 4A). We found 365 paths connect proteins in the p53 network to proteins in the NF-κB network; among which, only two are direct connections and 295 require a connector protein. The two direct interactions were revealed to be between: P53_HUMAN and IKKA_HUMAN, and P53_HUMAN and IKBA_HUMAN proteins; illustrating potential connection route to consider when creating a unified model of the NF-κB and p53 system. Indirect links for the rest of the nodes were found to require protein mediators to act as connector proteins. The proteins acting as connectors between the two networks are shown in blue in Figure 4A, B and 4C. It is evident that the P53_HUMAN protein can itself act as a connecting protein between members of the NF-κB pathway and members of the p53 system (*for example*, CDN1A_HUMAN - P53_HUMAN - IKKA_HUMAN; and, MDM2_HUMAN - P53_HUMAN - IKKA_HUMAN).

After having determined the shortest paths linking the p53 and NF-κB systems, the identified connector proteins linking the two systems were grouped according to their frequency values, and cross referenced with reference databases, for the interpretation of their functional properties. Table 8 provides a list of the top ten connecting nodes with the most number of paths passing through it [see Additional file 1: Suppl. 14-18 for further information extracted for these proteins]. It shows the frequency values for the connector protein with shortest paths *l *= 2, and the biological processes associated with the connector protein. Members of the NF-κB and p53 network sharing the same connector protein are also listed in Table 8 (Note, only two examples for each connection with the same frequency values have been presented). Interestingly, Heat shock protein HSP 90-beta (HS90B_HUMAN) and Ubiquitin (UBIQ_HUMAN) were revealed to be important proteins with the highest frequency, *f *= 15, linking proteins in the p53 system to proteins in the NF-κB system. Two examples of each connection are: CD2A2_HUMAN - *HS90B_HUMAN *- TF65_HUMAN; and P53_HUMAN - *HS90B_HUMAN *- IKBB_HUMAN for HSP 90-beta; and MDM2_HUMAN - UBIQ_HUMAN - RELB_HUMAN; and CDN1A_HUMAN - UBIQ_HUMAN - NFKB2_HUMAN for Ubiquitin. The frequency results were useful in establishing the importance of shared proteins between systems.

**Table 8:** Frequency and *Biological process *information on NF-κB and p53 networks connectors

*Connector protein*	*Frequency*	*Biological process involved*	*Protein in p53*	*Protein in NF-κB*.
Heat shock protein HSP 90-beta (HS90B_HUMAN)	15		CD2A2_HUMAN	TF65_HUMAN
			P53_HUMAN	IKBB_HUMAN

Ubiquitin (UBIQ_HUMAN)	15	13 processes]: APC-Cdc20 mediated degradation of Nek2A; APC/C:Cdh1-mediated degradation of Skp2; Apoptosis; Cdc20:Phospho-APC/C mediated degradation of Cyclin A; Cell Cycle Checkpoints; Cell Cycle, Mitotic; DNA Replication; HIV Infection; Regulation of activated PAK-2p34 by proteasome mediated degradation; Signalling by EGFR; Signalling by Wnt; Signalling in Immune system; Signalling by NGF	MDM2_HUMAN	RELB_HUMAN
			CDN1A_HUMAN	NFKB2_HUMAN

Poly [ADP-ribose] polymerase 1 (PARP1_HUMAN)	10		CDN1A_HUMAN	NFKB1_HUMAN
			P53_HUMAN	IKKB_HUMAN

Stress-70 protein, mitochondrial (GRP75_HUMAN)	9		P53_HUMAN	REL_HUMAN
			P53_HUMAN	NFKB1_HUMAN

Heat shock cognate 71 kDa protein (HSP7C_HUMAN)	9	Membrane Trafficking	P53_HUMAN	IKBE_HUMAN
			P53_HUMAN	IKKE_HUMAN

CREB-binding protein (CBP_HUMAN)	8	Gene Expression	MDM2_HUMAN	IKKA_HUMAN
			MDM2_HUMAN	IKKB_HUMAN

Immunoglobulin heavy chain-binding protein (GRP78_HUMAN)	8	Hemostasis	P53_HUMAN	NFKB1_HUMAN
			P53_HUMAN	NFKB2_HUMAN

Heat shock 70 kDa protein 1L (HS71L_HUMAN)	8		P53_HUMAN	REL_HUMAN
			P53_HUMAN	RELB_HUMAN

Heat shock protein HSP 90-alpha (HS90A_HUMAN)	7		P53_HUMAN	IKBB_HUMAN
			P53_HUMAN	IKBE_HUMAN

Nuclear receptor subfamily 3 group C member 1 (GCR_HUMAN)	7	Gene Expression	MDM2_HUMAN	NFKB1_HUMAN
			MDM2_HUMAN	NFKB2_HUMAN

### Network of interactions between p53, NF-κB and the G1/S phase of the Cell cycle

**Figure 5:**
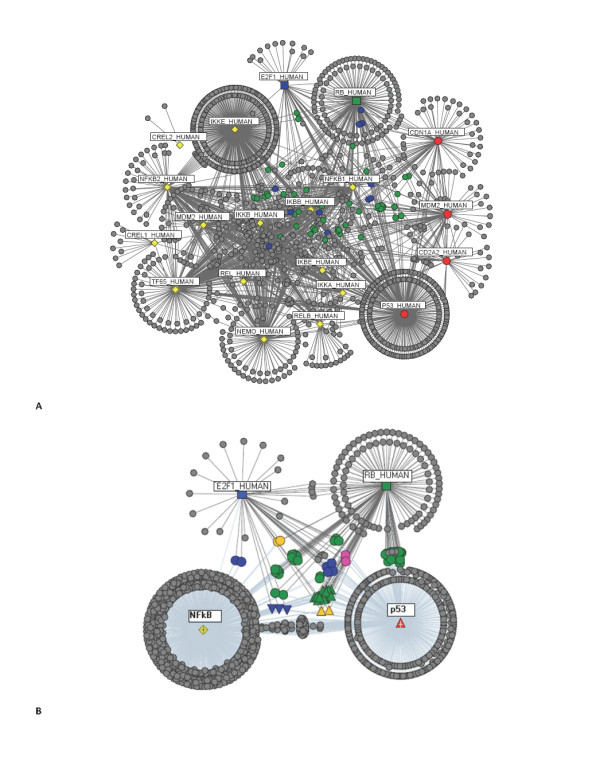
**p53 and NF-κB with RB_HUMAN and E2F1_HUMAN**. **(A) **Members of p53 (red circles) and NF-κB (yellow diamonds) networks, their connections with RB_HUMAN (green square) and E2F1_HUMAN (blue square}) cell cycle proteins, and the common components shared between them. Components connecting RB_HUMAN with p53 and NF-κB networks are denoted in green, whilst the components connecting E2F1_HUMAN with the two networks are denoted in blue. **(B) **A condensed view of only the p53 and NF-κB networks, and their interactions with RB_HUMAN and E2F1_HUMAN proteins. Triangular connector nodes represent common components between RB_HUMAN and the two networks (in green), E2F1_HUMAN and the two networks (in blue), and RB_HUMAN and E2F1_HUMAN connections with the NF-κB and p53 networks (in yellow). Circular nodes in green denote RB_HUMAN connectors to p53 or NF-κB networks; and in blue for E2F1_HUMAN to p53 or NF-κB networks. The yellow and magenta circular nodes represent proteins connecting both E2F1_HUMAN and RB_HUMAN to members of the NF-κB (in yellow) and p53 (in magenta). Refer also to Tables 9, 10, 11, 12 and 13 for further information.

Since it has been suggested, that some cell cycle proteins are activated by one pathway and are relevant for the regulation of another [44,65-69], it was of interest to investigate the relationship between the NF-κB, p53 and the cell cycle systems. For this study, only events leading to the G1/S transition phase of the cell cycle, the point where NF-κB and p53 signal transduction events are active the most [93] were considered. We start by exploring the interactions between RB_HUMAN and E2F1_HUMAN cell cycle proteins, with members of the p53 and NF-κB networks. Figure 5 show the network obtained from this analysis. Proteins that link the proteins in the p53 and NF-κB networks to RB_HUMAN are denoted in green, whilst the proteins connecting the two networks to E2F1_HUMAN are in blue (Figure 5A). Common protein shared between the p53 and NF-κB networks have been represented in the form of green triangles (for links to RB_HUMAN) and blue triangles (for links with E2F1_HUMAN) (see Figure 5B). Closer evaluation of the interactions linking the p53 network to the cell cycle proteins (Table 9), identified 46 shortest paths for interactions with RB_HUMAN (44 of which are indirect links mediated by a single node and 2 direct links {CDN1A_HUMAN - RB_HUMAN [66,94]; MDM2_HUMAN - RB_HUMAN }); and 19 shortest paths for interactions with E2F1_HUMAN (17 of which are indirect links mediated by a single node, and 2 direct links {CD2A2_HUMAN - E2F1_HUMAN; P53_HUMAN - E2F1_HUMAN }). These results therefore suggest an active role of CDN1A_HUMAN (p21) and MDM2_HUMAN (mdm2) on the activity of the RB_HUMAN protein in the cell cycle. And thus implies possible connection routes to consider when constructing a unified model of the p53 and the G1/S phase of the cell cycle networks. Likewise, for the NF-κB network, 74 shortest paths were identified linking NF-κB proteins to RB_HUMAN (all of which were indirect links); and 36 shortest paths for interactions with E2F1_HUMAN (of which only a single direct link was observed, IKBA_HUMAN - E2F1_HUMAN).

**Table 9:** Shortest paths

p53 network and cell cycle proteins				
	**Rb**	**E2F-1**	**P27**	**Cyclin D1**

Direct link (*l *= 1)	2	2	0	1
Path with *l *= 2	44	17	31	25
Total shortest paths of all length *l *(*l *= 1, *l *= 2, *l *= 3, *l *= 4, ...)	46	19	31	26

				

**NF-κB network and cell cycle proteins**				

				

	Rb	E2F-1	P27	Cyclin D1

Direct link (*l *= 1)	0	1	0	0
Path with *l *= 2	72	35	33	46
Total shortest paths of all length *l *(*l *= 1, *l *= 2, *l *= 3, *l *= 4, ...)	74	36	83	91

Path lengths with *l *= 1 is said to be a direct link. Total shortest path length is where *l *= 1, *l *= 2, *l *= 3 and *l *= 4

**Figure 6:**
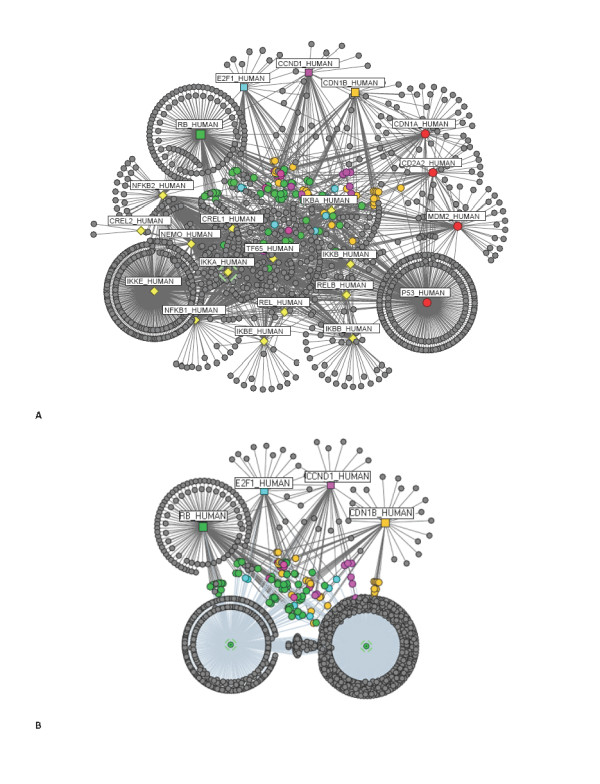
**Network representation of p53, NF-κB and cell cycle interactions**. **(A) **Network topology of the combined networks of the p53 (red), NF-κB (yellow) and the cell cycle {CDN1B_HUMAN (orange), CCND1_HUMAN (magenta), RB_HUMAN (green), E2F1_HUMAN (blue)}. Connector nodes linking cell cycle proteins to either NF-κB or p53; or to both have been denoted according to the colour of the cell cycle protein counterpart. For example, since E2F1_HUMAN is denoted in blue, connector proteins linking it to the p53 or NF-κB, or to both will be highlighted in blue **(B) **Condensed view of the p53 and NF-κB networks, and their connections with cell cycle proteins. The connectors have been labelled according to **(A)**.

We repeated this analysis to include interactions between the rest of the G1/S cell cycle proteins (RB_HUMAN, CCND1_HUMAN, CDN1B_HUMAN, and E2F1_HUMAN) and the members of the p53 and NF-κB networks (see Figure 6 - only the connecting nodes linking CDN1B_HUMAN *(p27, circle, yellow)*, CCND1_HUMAN *(Cyclin D1, circle, magenta)*, RB_HUMAN (*Rb, circle, green) *and E2F1_HUMAN *(E2F-1, circle, blue) *to the p53 and NF-κB networks have been colour coded - Figure 6A and 6B). Shortest path lengths calculated for interactions between proteins in the p53 network and CDN1B_HUMAN, numbered 31 (all indirect links with path length = 2); and 26 for interactions with CCND1_HUMAN (25 indirect connection with path length = 2 and 1 direct connection {CDN1A_HUMAN - CCND1_HUMAN}). Similarly, for the NF-κB system, 83 shortest paths connecting CDN1B_HUMAN (33 of which have path length = 2), and 91 shortest paths connecting CCND1_HUMAN(46 of which are indirect links mediated by a single connector path length = 2) to members of the NF-κB network were determined (see Table 9 for shortest paths statistics). Frequency values and functional properties ascertained for nodes linking the p53 and cell cycle networks, as well for those linking the NF-κB with the cell cycle network have been reviewed in Table 10, 11, 12 and 13 [see Additional file 1: Suppl. 19-24 for a full list].

**Table 10:** Frequent components linking E2F1_HUMAN to NF-κB pathway

*Connector protein*	*Frequency*	*Protein in NF-κB*	*Additional information of connector proteins*
NFKB1_HUMAN	9	IKBA_HUMAN	Nuclear factor NF-kappa-B p105 subunit
PARP1_HUMAN	4	NFKB2_HUMAN	Poly [ADP-ribose] polymerase 1
NCOA3_HUMAN	3	IKKA_HUMAN	Nuclear receptor coactivator 3
CUL1_HUMAN	3	IKBB_HUMAN	Cullin-1
CBP_HUMAN	3	TF65_HUMAN	CREB-binding protein (Involved in Gene Expression process)
SP1_HUMAN	2	TF65_HUMAN REL_HUMAN	Transcription factor Sp1
P53_HUMAN	2	IKKA_HUMAN	Cellular tumor antigen p53
TIP60_HUMAN	1	CREL1_HUMAN	Histone acetyltransferase HTATIP
PHB_HUMAN	1	TF65_HUMAN	Prohibitin
PA2G4_HUMAN	1	IKKE_HUMAN	Proliferation-associated protein 2G4

**Table 11:** Frequent components linking RB_HUMANto NF-κB pathway

*Connector protein*	*Frequency*	*Protein in NF-κB*	*Additional information of connector proteins*
HSP7C_HUMAN	10	IKKB_HUMAN	Heat shock cognate 71 kDa protein (Involved in Membrane Trafficking process)
HDAC2_HUMAN	4	NFKB1_HUMAN	Histone deacetylase 2 (Involved in 2 processes: Gene Expression; Signalling by NGF)
ESR1_HUMAN	3	IKKB_HUMAN	Estrogen receptor
HDAC1_HUMAN	3	IKKA_HUMAN	Histone deacetylase 1 (Involved in 2 processes: Gene Expression; Signalling by NGF)
SMCA4_HUMAN	3	RELB_HUMAN	Probable global transcription activator SNF2L4
TBP_HUMAN	3	NFKB2_HUMAN	TATA-box-binding protein (Involved in 3 processes: Gene Expression; HIV Infection; Transcription)
BRCA1_HUMAN	2	TF65_HUMAN	Breast cancer type 1 susceptibility protein (Involved in DNA Repair process)
ANDR_HUMAN	2	TF65_HUMAN	Androgen receptor
CEBPB_HUMAN	2	NFKB1_HUMAN	CCAAT/enhancer-binding protein beta
CDK9_HUMAN	2	TF65_HUMAN	Cell division protein kinase 9 (Involved in 9 processes: Elongation arrest and recovery; Gene Expression; HIV Infection; HIV-1 elongation arrest and recovery; Pausing and recovery of HIV-1 elongation; Pausing and recovery of Tat-mediated HIV-1 elongation; Pausing and recovery of elongation; Tat-mediated HIV-1 elongation arrest and recovery; Transcription)

**Table 12:** Frequent components linking E2F1_HUMAN to the p53 network

Connector protein	Frequency	Protein in p53	Additional information *of connector proteins*
P53_HUMAN	2	CDN1A_HUMAN	Cellular tumor antigen p53
CBP_HUMAN	2	MDM2_HUMAN	CREB-binding protein
RB_HUMAN	2	CDN1A_HUMAN	Retinoblastoma-associated protein
TIP60_HUMAN	1	MDM2_HUMAN	Histone acetyltransferase HTATIP
SKP2_HUMAN	1	CDN1A_HUMAN	S-phase kinase-associated protein 2
PARP1_HUMAN	1	CDN1A_HUMAN	Poly [ADP-ribose] polymerase 1
ATM_HUMAN	1	MDM2_HUMAN	Serine-protein kinase ATM (Involved in 2 processes: Cell Cycle Checkpoints; DNA Repair)
MDM4_HUMAN	1	MDM2_HUMAN	Protein Mdm4
CHK2_HUMAN	1	MDM2_HUMAN	Serine/threonine-protein kinase Chk2 (Involved in 3 processes: Cdc20:Phospho-APC/C mediated degradation of Cyclin A; Cell Cycle)
CDK3_HUMAN	1	CDN1A_HUMAN	Cell division protein kinase 3

**Table 13:** Frequent components linking RB_HUMAN to the p53 network

Connector protein	Frequency	Protein in p53	Additional information *of connector proteins*
P53_HUMAN	2	CDN1A_HUMAN	Cellular tumor antigen p53
CBP_HUMAN	2	MDM2_HUMAN	CREB-binding protein
RB_HUMAN	2	CDN1A_HUMAN	Retinoblastoma-associated protein
TIP60_HUMAN	1	MDM2_HUMAN	Histone acetyltransferase HTATIP
SKP2_HUMAN	1	CDN1A_HUMAN	S-phase kinase-associated protein 2
PARP1_HUMAN	1	CDN1A_HUMAN	Poly [ADP-ribose] polymerase 1
ATM_HUMAN	1	MDM2_HUMAN	Serine-protein kinase ATM (Involved in 2 processes: Cell Cycle Checkpoints; DNA Repair)
MDM4_HUMAN	1	MDM2_HUMAN	Protein Mdm4
CHK2_HUMAN	1	MDM2_HUMAN	Serine/threonine-protein kinase Chk2 (Involved in 3 processes: Cdc20:Phospho-APC/C mediated degradation of Cyclin A; Cell Cycle)
CDK3_HUMAN	1	CDN1A_HUMAN	Cell division protein kinase 3

## Conclusion

A network is usually thought of as a coherent system that comprises of units interacting in some kind of orchestrated and regulated fashion - such that the emergent behaviour of the whole (i.e. the network) is recognisable and can be characterised. Once some of the behaviour is recognised, the system can be described at a level of detail appropriate to the system's behaviour whilst ignoring the details of the constituent parts. Since molecular networks are large and complex, with their components and their interactions quite heterogeneous characterising the relationship between structure and dynamics of the system makes it far from straightforward. Although research aiming at coping with these challenges has become very popular, it is important to bear in mind that the current efforts can only profit from a combined theoretical and experimental approach. This is where the approach presented in this paper becomes beneficial. The idea is that by combining both the data driven and knowledge driven strategies, direct and or combinatorial interaction parameters of many protein can be captured from the information gained, and can thus be used to construct, guide and or unify dynamical models of signal transduction pathways from which a realistic model of the systems behaviour can be determined. The resulting dynamical model can then provide the conceptual and explanatory linkage between the observed phenomena and the predicted.

This framework of computational modelling of molecular networks at various levels or organisation has the potential to allow cost effective experimentation and hypothesis exploration, computationally uncovering the behaviour of molecular species and combinatorial interactions that would be difficult and too expensive to carry out in a wet-lab setting. While, network topology analysis is thus useful for showing which proteins in the network depend on which other protein, it does not give us any further information on the regulatory effects of these dependencies. Despite these methodological limitations, our results offer a view, demonstrating the importance of elucidating the functional roles key or shared components play in the propagation of signals across transduction systems.

The main implication of the presented application is the recognition that changes in one signalling system, undoubtedly causes a ripple effect on the rest of the surrounding system - as shown by the extensive interconnection of the systems studied and their common shared components. It is hoped that the use of this form of analysis may also be beneficial in highlighting areas of research where very little is known for further future study.

## Authors' contributions

AECI conceived the project and design. AECI and TPN prepared the data. TPN extracted processed data from public databases, implemented the algorithms and analysed the results. AECI and TPN wrote the paper. All authors read and approved the document.

## Supplementary Material

Additional file 1 **Supplementary Material**. The data provided correspond to supplementary calculation details, as well as additional information on the topological and functional properties of the p53, NF-κB and cell cycle networks. All proteins are listed according to their *Uniprot *accession number and *protein *ID name. Click here for file
